# PARP Inhibitors Display Differential Efficacy in Models of *BRCA* Mutant High-Grade Serous Ovarian Cancer

**DOI:** 10.3390/ijms22168506

**Published:** 2021-08-07

**Authors:** Kristie-Ann Dickson, Tao Xie, Christian Evenhuis, Yue Ma, Deborah J. Marsh

**Affiliations:** 1 Translational Oncology Group, School of Life Sciences, Faculty of Science, University of Technology Sydney, Ultimo, NSW 2007, Australia; Kristie-Ann.Dickson@uts.edu.au (K.-A.D.); Tao.Xie-1@alumni.uts.edu.au (T.X.); Yue.Ma-7@student.uts.edu.au (Y.M.); 2 iThree Institute, School of Life Sciences, Faculty of Science, University of Technology Sydney, Ultimo, NSW 2007, Australia; christian.evenhuis@gmail.com; 3 Northern Clinical School, Faculty of Medicine and Health, University of Sydney, Camperdown, NSW 2006, Australia

**Keywords:** BRCA1, BRCA2, homologous recombination repair, PARP inhibitor, olaparib, rucaparib, niraparib, talazoparib, veliparib

## Abstract

Several poly (adenosine diphosphate-ribose) polymerase (PARP) inhibitors are now in clinical use for tumours with defects in BReast CAncer genes *BRCA1* or *BRCA2* that result in deficient homologous recombination repair (HRR). Use of olaparib, niraparib or rucaparib for the treatment of high-grade serous ovarian cancer, including in the maintenance setting, has extended both progression free and overall survival for women with this malignancy. While different PARP inhibitors (PARPis) are mechanistically similar, differences are apparent in their chemical structures, toxicity profiles, PARP trapping abilities and polypharmacological landscapes. We have treated ovarian cancer cell line models of known *BRCA* status, including the paired cell lines PEO1 and PEO4, and UWB1.289 and UWB1.289+BRCA1, with five PARPis (olaparib, niraparib, rucaparib, talazoparib and veliparib) and observed differences between PARPis in both cell viability and cell survival. A cell line model of acquired resistance to veliparib showed increased resistance to the other four PARPis tested, suggesting that acquired resistance to one PARPi may not be able to be rescued by another. Lastly, as a proof of principle, HRR proficient ovarian cancer cells were sensitised to PARPis by depletion of *BRCA1*. In the future, guidelines will need to emerge to assist clinicians in matching specific PARPis to specific patients and tumours.

## 1. Introduction

The advent of pharmacological inhibitors of the DNA repair enzyme poly (adenosine diphosphate-ribose) polymerase (PARP) has heralded major therapeutic advances for malignancies that have defects in components of homologous recombination repair (HRR) pathways [1,2,3]. The focus of PARP inhibitors (PARPis) to date has been on *BRCA1* and *BRCA2* mutated tumours, with clinical benefits seen in patients with mutations in these DNA repair genes such as ovarian [4], breast [5], prostate [6] and pancreatic cancers [7]. *BRCA1* methylated tumours are also sensitive to PARP inhibition [8], as are tumours with mutations in other genes that function in repair of double strand breaks (DSBs), including *RAD51C*, *RAD51D, ATM* and *PALB2*, where tumours are described as having a “BRCAness” phenotype [8,9,10]. With this broadening concept of BRCAness, other malignancies are being investigated to assess sensitivity to PARP inhibitors, including colorectal, upper gastrointestinal and acute myeloid leukemia [1,11,12,13].

PARP family members function in a number of cellular processes including the regulation of gene transcription, chromatin remodelling and DNA repair [14,15]. PARPs bind to DNA at sites of single strand breaks (SSBs) undergoing base excision repair (BER) where they function to recruit DNA repair machinery. When the replication fork comes across a SSB, DSBs are generated that then need to undergo HRR [16,17]. BER is the default repair pathway for cells with defects in HRR, the preferred pathway for repair of DSBs, such as occurs in the presence of *BRCA* mutations. By inhibiting PARP function in cells with deficient HRR, cells lose their ability to choose the default BER pathway to repair DNA damage, creating synthetic lethality that leads to cell death [18,19,20].

PARP1, PARP2, PARP3, PARP4 (also known as Vault PARP) and tankyrases 1 and 2 (PARP5a and PARP5b) are amongst the most studied members of the PARP family [2,15]. Catalytic activation of PARP1 synthesises poly (ADP-ribose), PAR, from the substrate nicotinamide adenine dinucleotide (NAD^+^) in a process known as PARylation. While PARP1 is reported to conduct more than 90% of PARylation associated with DNA damage, PARPs 2, 3, 4, 5a and 5b also demonstrate PARylation activity [21,22]. Inhibition of these PARP enzymes and in turn the PARylation process has proven to be a major advancement in the treatment of HRR deficient tumours [1,2,3,23,24].

Olaparib (Lynparza^®^) was the first PARPi endorsed by the Food and Drug Administration (FDA) for the treatment of advanced germline *BRCA*-mutated ovarian cancer in 2014, followed by rucaparib (Rubraca^®^) for use to treat the same indication in 2016 [18,25,26,27]. Olaparib and rucaparib were sanctioned in 2018 for use as maintenance therapy for women with ovarian cancer following surgery. Niraparib (Zejula^®^) was endorsed in 2020 by the FDA as maintenance for advanced epithelial ovarian, fallopian tube or primary peritoneal cancer where patients have had complete or partial response to first-line platinum-based chemotherapy [28]. Talazoparib (Talzenna^®^) became licensed by the FDA in 2018 for the treatment of *BRCA*-mutated HER2-negative breast cancers [29]. Veliparib (ABT-888) is one of a number of PARPis that have not been endorsed to date for mainstream clinical use. Each of these PARPis has a unique structure and different binding affinities for PARP family members [25,30]. Furthermore, these PARPis display differential PARP trapping potencies, where the PARP complex locks onto or becomes trapped on DNA at the site of breakage, thus preventing binding of other DNA repair factors [17,31]. The PARP trapping potency of these five PARPis from highest to lowest is talazoparib, niraparib, rucaparib, olaparib, then veliparib [24]. While amongst the most efficacious molecular target drugs of recent times, tumours can display innate or acquired resistance to PARPis. The reasons for this include innate HRR proficiency, reversion of *BRCA* mutations or mutations in other HRR-related genes such as *PALB2* or *RAD51C*, loss of *BRCA1* methylation that re-establishes HRR proficiency, the increase in expression of drug efflux pumps such as the MDR1 (p-glycoprotein) gene, aberrant replication fork protection and down-regulation of PARP proteins themselves, possibly as a result of PARP trapping [32,33].

Here, we have focused on ovarian cancer, where over 50% of the most common sub-type high-grade serous ovarian cancer (HGSOC) have defects in genes that function in HRR [32]. We use models of *BRCA* wild-type and mutant ovarian cancer to investigate the efficacy of five PARPis—olaparib, rucaparib, niraparib, talazoparib and veliparib—on cell viability and cell survival. Further, we have investigated whether acquired PARP resistance to veliparib can be overcome by use of other PARPis. Lastly, we manipulated HRR by down-regulating *BRCA1* in *BRCA1* wild-type cells, including in OVCAR-3 cells known to harbor a *CCNE1* amplification and be HRR proficient, to determine whether we could sensitise cells to PARP inhibitors.

## 2. Results

### 2.1. PARPis Display Differential Efficacy on Cell Viability in BRCA Wild-Type and Mutant Ovarian Cancer Cell Line Pairs

**Figure 1:**
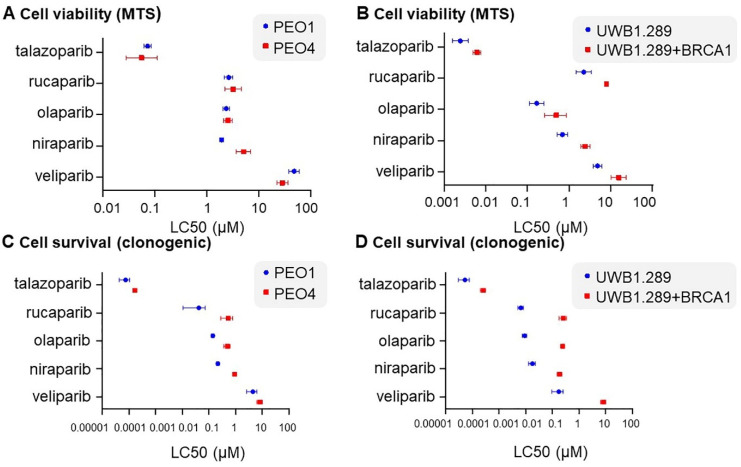
LC50 calculated from cell viability (MTS assay) data for each of five PARPis (talazoparib, rucaparib, olaparib, niraparib and veliparib) in (**A**) PEO1 and PEO4; (**B**) UWB1.289 and UWB1.289+BRCA1. LC50 calculated from cell survival (clonogenic assay) data for the identical five PARPis in (**C**) PEO1 and PEO4; (**D**) UWB1.289 and UWB1.289+BRCA1.

LC50 levels of five PARPis (olaparib, niraparib, rucaparib, talazoparib and veliparib) in PEO1 and PEO4 (Figure 1A), as well as UWB1.289 and UWB1.289+BRCA1 cells (Figure 1B), were determined from dose curves of each drug and endpoint MTS assay (Figures S1 and S2). Doses and serial dilutions used for each PARPi in different cell lines for all experiments are summarised in Table S1. *BRCA2* mutant PEO1 cells were responsive to olaparib, niraparib and talazoparib over the dose curve compared with wild-type (WT) PEO4 cells, but not to rucaparib and veliparib in the context of cell viability (Figure S1). Veliparib displayed the highest of all LC50s at 47.59 µM in PEO1 cells and 28.13 µM in PEO4, suggesting that the PEO1/PEO4 cell line pair are highly resistant to this PARPi. Both WT PEO4 and the mutant PEO1 cell pair were highly sensitive to talazoparib (LC50s of 0.0557 and 0.0729 µM, respectively), suggesting that this sensitivity was independent of *BRCA2* status (Table 1).

*BRCA1* mutant UWB1.289 cells were more responsive to all five PARPis tested based on LC50 data, compared to the paired cell line UWB1.289+BRCA1 (Table 1). Similar to the PEO paired cell lines, the UWB1.289 paired lines displayed the greatest sensitivity to talazoparib (Table 1). Niraparib showed the greatest discrimination in both cell line pairs based on the largest fold change in LC50 between mutant and WT cells, followed by rucaparib (Table 1). Further, greater fold changes based on LC50 data in response to all PARPis were seen in the *BRCA1* mutant and WT pair UWB1.289 compared with the *BRCA2* mutant and WT pair PEO1 and PEO4 (Table 1). This suggests that different *BRCA* mutations may respond differently to a range of PARPis.

### 2.2. PARPis Display Differential Efficacy on Cell Survival in BRCA Wild-Type and Mutant Ovarian Cancer Cell Line Pairs

LC50 doses calculated using cell survival data from clonogenic assays of each PARPi in *BRCA* WT and mutant pairs were determined (Table 2, Figure 1C,D). As expected, greater sensitivity to all PARPis was seen in the *BRCA* mutant cell line of each pair (PEO1 and PEO4, Figure S3; UWB1.289 and UWB1.289+BRCA1, Figure S4) in the context of cell survival. As for cell viability, based on LC50 doses, the greatest sensitivity was observed for talazoparib in all cell lines (Table 2). Again, as for cell viability, differences in cell survival post treatment with different PARPis were seen in both of the cell line pairs tested. Veliparib showed the greatest fold change in the UWB1.289 pair, with a 47.36-fold difference in LC50 between the mutant and WT cell lines (Table 2). Curiously, veliparib showed the least fold difference in LC50 values between the mutant and WT PEO pair, with rucaparib showing the largest fold change at 12.78 (Table 2). Overall, greater fold differences in all PARPis were observed in the UWB1.289 pair compared to the PEO pair (Table 2). This observation was also true for cell viability (Table 1).

### 2.3. Veliparib Resistant A2780 Cells Retain Resistance to Other PARP Inhibitors

**Figure 2:**
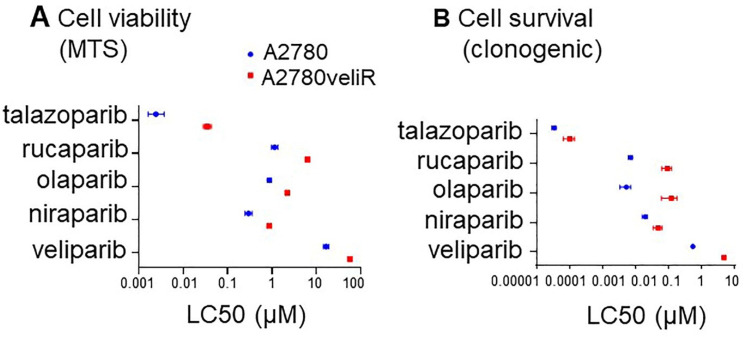
Talazoparib, rucaparib, olaparib or niraparib cannot rescue acquired veliparib resistance in the A2780veliR cell line model. (**A**) LC50 calculated from cell viability (MTS assay) data post treatment with a PARPi. (**B**) LC50 calculated from cell survival (clonogenic assay) data post treatment with a PARPi.

Given that different PARPis target PARP family members with varying efficiency, as well as show differences in their PARP trapping potency, we sought to determine whether olaparib, niraparib, talazoparib or rucaparib could rescue acquired resistance to veliparib. The A2780veliR cell line was developed in-house by the addition of increasing doses of ABT-888 and based on LC50 dose calculated from MTS assay was found to be 3.46-fold more resistant to veliparib than parental A2780 cells (Table 1, Figure 2A and Figure S5). Furthermore, based on LC50 doses calculated from cell survival data, A2780veliR cells were 8.84-fold more resistant to veliparib than A2780 (Table 2, Figure 2B and Figure S6). A2780veliR cells were between 2.54- to 14.41-fold more resistant to the other PARPis tested than parental A2780 cells based on LC50 doses calculated from MTS data (Table 1, Figure 2A and Figure S5). Furthermore, LC50 levels calculated from clonogenic assays showed that A2780veliR cells were between 2.49- to 23.03-fold more resistant to the other PARPis tested (Table 2, Figure 2A and Figure S6). These data support the conclusion that acquired veliparib resistance *in vitro* also leads to greater resistance to other PARPis. It therefore seems unlikely that PARPis will be able to rescue acquired resistance to a specific PARPi, at least in the case of resistance to veliparib.

### 2.4. Down-Regulation of BRCA1 in BRCA1 Wild-Type Ovarian Cancer Cell Lines Sensitises Cells to PARPis

**Figure 3:**
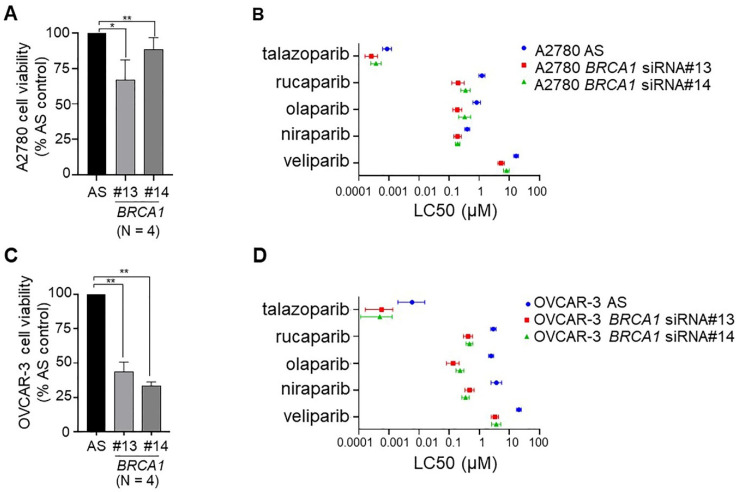
Down-regulation of *BRCA1* decreased basal cell viability and sensitised cells to PARP inhibition. Down-regulation of *BRCA1* by two distinct siRNAs (#13 and #14) in (**A**) A2780 cells and (**C**) OVCAR-3 cells decreased cell viability measured by MTS assay (N = 4; AS, AllStars control siRNA). LC50 calculated from cell viability (MTS assay) data for each of the five PARPis (talazoparib, rucaparib, olaparib, niraparib and veliparib) after *BRCA1* down-regulation in (**B**) A2780 cells and (**D**) OVCAR-3 cells. * *p* < 0.05, ** *p* < 0.01.

Next, we sought to determine whether we could sensitise ovarian cancer cells to PARP inhibition by down-regulation of a key component of HR, specifically *BRCA1*. We chose two *BRCA1* WT cell lines for this purpose, specifically OVCAR-3 with a known *CCNE1* amplification frequently associated with HR proficiency [34], and A2780 that has been speculated to harbor a defect in DNA repair [35]. *BRCA1* was down-regulated using two independent siRNAs. We achieved between 56 and 62% *BRCA1* down-regulation in OVCAR-3 cells and 40–53% down-regulation in A2780 cells (Figure S7). Down-regulation of *BRCA1* alone in both cell lines decreased cell viability, by 12–33% in A2780 cells and 56–67% in OVCAR-3 cells (Figure 3). The large decrease in cell viability for OVCAR-3 cells upon down-regulation of *BRCA1* is likely due to the presence of a *CCNE1* amplification, previously reported as mutually exclusive events [36]. Down-regulation of *BRCA1* in A2780 cells (Figure 3B and Figure S8) and OVCAR-3 cells (Figure 3D and Figure S9) lowered the LC50 dose for all five PARPis, indicating that loss of *BRCA1* in these cell lines sensitised them to PARP inhibitors (Table 3).

### 2.5. Down-Regulation of BRCA1 in BRCA1 Wild-Type Ovarian Cancer Cell Lines Decreases Cell Survival

**Figure 4:**
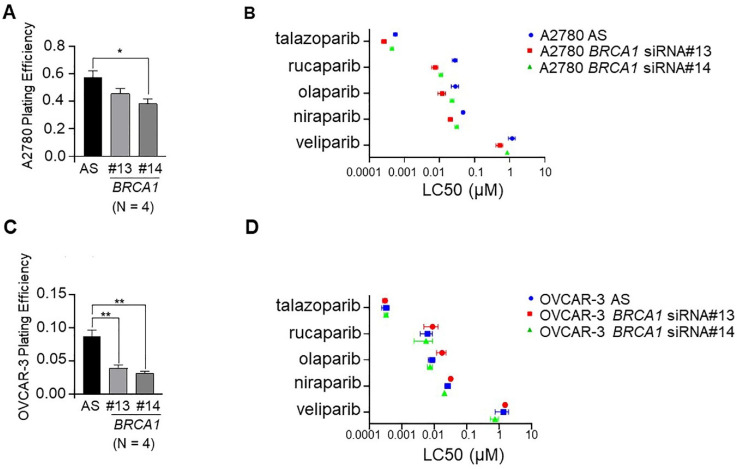
Down-regulation of *BRCA1* decreased basal cell survival, and in some cases, sensitised cells to PARP inhibition. Down-regulation of *BRCA1* by two distinct siRNAs (#13 and #14) in (**A**) A2780 cells and (**C**) OVCAR-3 cells decreased plating efficiency (N = 4; AS, AllStars control siRNA). LC50 data calculated from cell survival (clonogenic assay) for each of the five PARPis (niraparib, olaparib, rucaparib, talazoparib and veliparib) after *BRCA1* down-regulation in (**B**) A2780 cells and (**D**) OVCAR-3 cells. * *p* < 0.05, ** *p* < 0.01.

We then sought to determine the effect of down-regulation of *BRCA1* on cell survival in A2780 and HR proficient OVCAR-3 cells. In both cases, down-regulation of *BRCA1* caused a decrease in plating efficiency, with a greater decrease seen in OVCAR-3 cells (Figure 4C) than A2780 cells (Figure 4A), indicating a significant basal effect on cell survival of down-regulating *BRCA1* in these cell line models. Dose curves of cell lines treated with PARPis did not show a significant difference between cells treated with the non-silencing control or either of two *BRCA1* siRNAs (Figures S10 and S11). This is possibly due to the fact that down-regulation of *BRCA1* alone in these cells had a large effect on cell survival and any additional effects of PARP inhibition were difficult to detect. With this in mind, small differences in LC50 dose comparing the non-silencing control to cell lines in which *BRCA1* was down-regulated were observed for most PARPis in both cell line models (Figure 4B,D, Table 3).

## 3. Discussion

The fundamental premise of targeting the cell’s DNA repair machinery has seen the development and rapid uptake of PARPis in the clinic. Specific improvements have been seen in both progression free and overall survival for women with HGSOC treated with a PARPi [37,38,39,40,41]. Still, currently there is no clear rationale regarding which PARPi to use, for which ovarian cancer patients beyond FDA endorsement of olaparib, rucaparib or niraparib when a mutation is present in *BRCA1* or *BRCA2* after first-line platinum-based chemotherapy or as maintenance therapy [30,42]. We show clear differences in response and sensitivity to different PARPis in our cell line models with known *BRCA* mutation status. Based on LC50 doses calculated from cell viability data, *BRCA2* mutant PEO1 cells were actually less sensitive to talazoparib and veliparib than their mutation reversion counterpart cell line, PEO4. In contrast, UWB1.289 cells lacking *BRCA1* were more sensitive to all five PARPis tested, with larger fold differences in LC50 doses observed between this cell line and its WT *BRCA1* partner line compared with the PEO1/PEO4 pair for all PARPis analysed. This larger fold difference in LC50 dose for the UWB1.289 pair compared to the PEO1/PEO4 pair was also observed for cell survival calculated from clonogenic assays for all PARPis analysed.

There are a number of possible explanations for these observations. It is possible that there are inherent differences in response to PARPis based on whether tumours have a *BRCA1* or *BRCA2* mutation. To date, few studies have explored this possibility, although differences in response to PARPis in prostate cancer have been reported based on whether the tumour was *BRCA1* or *BRCA2* mutated [43]. While PARPis are mechanistically similar in that they all interact with the substrate NAD^+^ to inhibit PARylation and so DNA repair, they also have a number of differences. PARPis have different chemical structures and also differ in their ability to trap PARP1 on DNA, with talazoparib having the strongest PARP trapping function, followed by niraparib, rucaparib, olaparib and lastly veliparib [17,24]. This is consistent with our data that indicate talazoparib is the most cytotoxic of the PARPis tested and veliparib the least. Polypharmacology has been reported for PARP inhibitors. For example, niraparib and rucaparib have also been found to inhibit some kinases including DYRK15, CDK16 and PIM3 that may be therapeutically useful if these kinases are aberrantly expressed in specific tumours [25,44]. We cannot exclude the possibility that aberrant regulation of certain members of the kinome in cell line models used in this study may have affected the response to specific PARPis independently of *BRCA* status.

While the mechanism of acquired resistance to veliparib is currently unknown in our A2780veliR cells developed in-house from the A2780 parental cell line, we sought to investigate whether this resistance could be overcome by treatment with an alternative PARPi. The rationale for this strategy was based in the knowledge that different PARPis have been shown to display differential affinity for PARP family members, as well as different PARP trapping abilities [17,24,25,30,31]. Our data show that increased resistance to veliparib was not able to be overcome by treatment with any of olaparib, niraparib, rucaparib or talazoparib. In fact, increased resistance to veliparib led to increased resistance to all the other PARPis tested and would suggest that employing alternative PARPis would not be a successful clinical strategy to overcome acquired resistance to a PARPi. Testing of cell lines with developed resistance to other PARPis would need to be undertaken to further explore this phenomenon. Current approaches to overcoming PARPi resistance include focus on the use of inhibitors of other participants in the DNA damage repair response such as the cell cycle checkpoint regulators ATR, WEE1 and CHK1/2 [45,46,47,48].

Lastly, given the success of PARPis for women with HR deficient ovarian cancer, there is a strong need to expand these benefits to women whose tumours are HR proficient. With this in mind, in order to drive cells towards an HR deficient phenotype, we conducted a proof-of-principle experiment where we down-regulated *BRCA1* in HR proficient OVCAR-3 cells, as well as in the A2780 cell line that is *BRCA* WT but has recently been suggested to have defective DNA repair [35]. We then treated cells with all five PARPis. OVCAR-3 cells have an amplification of *CCNE1* that has been reported as a mutually exclusive event to defective HR [36]. In spite of the combination of *CCNE1* amplification and loss of *BRCA1* likely leading to synthetic lethality that would explain the large decrease in basal cell viability in OVCAR-3 cells upon depletion of *BRCA1*, we did observe increased sensitivity to all PARPis in *BRCA1* down-regulated cells based on LC50 doses. This was not seen in cell survival assays, likely due to the effects of synthetic lethality. In A2780 cell lines that may already have defective DNA repair, increases in sensitivity to PARP inhibition was observed following down-regulation of *BRCA1* in both cell viability and cell survival assays. This observation warrants broader exploration and suggests that responses to PARPis may be amenable to further improvement by targeting of key participants in HRR, even in cells that may already suffer impaired levels of DNA repair.

With multiple PARPis available now for clinical use, and likely additional ones in development that will be endorsed for future use, stronger guidance will be required as to which PARPi to choose for a specific patient, considering factors such as tumour type, stage of disease, the involvement of HRR genes possibly down to the level of specific mutations, as well as off-target effects of different PARPis that may be efficacious. The routine incorporation of organoids generated from primary tumours or PDx models into the clinical management of patients would assist in streamlining the choice of PARPi that would best suit particular cases [49,50]. Pharmacological targeting of components of HRR in HR proficient tumours may increase the cohort of patients who currently experience the benefits of PARPi therapy beyond those whose tumours harbor defects in HRR pathways.

## 4. Materials and Methods

### 4.1. Cell Lines

The human HGSOC cell lines UWB1.289, UWB1.289+BRCA1 [51] and OVCAR-3 [52] were purchased from the American Type Culture Collection (ATCC, Virginia, USA; respectively, cat. #CRL-2945, #CRL-2946 and #HTB-161), while PEO1 and PEO4 were gifts originating from Dr Simon Langdon [53]. UWB1.289 (University of Washington-BRCA1-family 289) was derived from a recurrent human papillary serous ovarian cancer that contained the c.2594delC germline mutation in exon 11 of *BRCA1*, resulting in a premature STOP at codon 845 and a *BRAC1*-null phenotype. The corresponding WT *BRCA1* allele was also lost. UWB1.289+BRCA1 cells were created following stable transfection of WT *BRCA1*. These paired cell lines also have a mutation in *TP53*, specifically c.625delAG and loss of the *TP53* WT allele. PEO1 and PEO4 cells were derived from peritoneal ascites of the same patient who had a poorly differentiated serous adenocarcinoma. PEO1 cells were collected after the patient was treated with cisplatin, 5-fluorouracil and chlorambucil. PEO4 cells were collected after the patient demonstrated resistance to these drugs. PEO1 cells have the *BRCA2* mutation c.5193C>G, and PEO4 cells have a second mutation in *BRCA2*, c.5193C>T, that restores WT BRCA2 [54,55]. This cell line pair also has a mutation in *TP53*, c.731G>A. The endometroid ovarian cancer cell line A2780 was sourced from Sigma-Aldrich Pty. Ltd. (cat. #93112591, Sydney, NSW, Australia) [35,52]. Clear defects in components of HRR have not been identified in A2780 cells, although they have recently been reported to have low levels of the repair factor RAD50 compared with their counterpart cisplatin resistant line A2780cisR, suggesting they may harbour deficiencies in HRR [35]. Further, A2780 cells have previously been reported to exhibit sensitivity to a PARP inhibitor [56]. The A2780veliR cell line is resistant to the PARPi veliparib (ABT-888, cat. #ALX-270-444-M005, Sapphire Biosciences, Waterloo, NSW, Australia) and was developed in our laboratory from the parental A2780 cell line by exposure to gradual increasing concentrations of ABT-888 (10–140 μM) over a 31-week period. Cells were then grown for 6 weeks veliparib free to wash out any remaining drug.

All cell lines were grown in RPMI 1640 (cat. #42402016, Thermo Fisher Scientific, Mulgrave, VIC, Australia) supplemented with 10% FBS (AusGeneX, Molendinar, QLD, Australia), with the exception of the UWB1.289 and UWB1.289 + BRCA1 cell lines which were maintained in 50% RPMI 1640 (HyClone #SH30027, GE Healthcare Life Sciences), 50% MEGM (Clonetics™ MEBM supplemented with SingleQuot additives cat. #CC-3150 from LONZA, Walkersville, MD, USA), supplemented with 3% FBS at 37 °C in a humidified 5% CO_2_ atmosphere.

Cell line authentication was performed by the Australian Genome Research Facility (AGRF) Melbourne, Australia, by short tandem repeat (STR) profiling using the GenePrint-10 System which co-amplifies ten loci, including the ASN-0002 loci (TH01, TPOX, vWA, Amelogenin, CSF1PO, D16S539, D7S820, D13S317 and D5S818) as well as D21S11. All cell lines tested negative for mycoplasma with the MycoAlert^TM^ Mycoplasma Detection Kit (cat. #LT07-318, LONZA, Walkersville, MD, USA).

### 4.2. BRCA1 Down-Regulation, RNA Extraction and qRT-PCR

Cells were seeded into 6-well plates (600,000/well for A2780; 1,000,000/well for OVCAR-3) for 24 h followed by transfection with 20 nM *BRCA1* siRNA #13 (cat. #SI02654575, Qiagen (cat. #301707), Chadstone, VIC, Australia), *BRCA1* siRNA #14 (cat. #SI02664361, Qiagen) or a non-silencing negative control (Allstars, Qiagen) using HiPerfect transfection reagent (Qiagen). After 18 h, siRNA transfected cells were re-seeded for MTS or clonogenic assays. RNA was extracted 48 h post transfection using the RNeasy Mini kit (cat. #74106, Qiagen) and 500 ng converted to cDNA using the SuperScript™ IV First-Strand Synthesis System (SSIV, cat. #18091200, Thermo Fisher Scientific Australia Pty. Ltd., Scoresby, VIC, Australia). Quantitative real-time PCR (qRT-PCR) was performed using the TaqMan Fast Advanced Master Mix Kit (cat. #444557, Thermo Fisher Scientific) with Taqman assays *BRCA1* (cat. #Hs01556193_m1, Thermo Fisher Scientific) and *hydroxymethylbilane synthase* (*HMBS)* endogenous control (cat. #97639748, Integrated DNA Technologies, Baulkham Hills, NSW, Australia) on the QuantStudio 12K Flex Real-Time PCR System (Thermo Fisher Scientific). Each experiment was performed in triplicate and repeated at least three times, with data reported as the mean ± S.E.M.

### 4.3. Cell Viability Assays and Calculation of LC50 Doses for PARP Inhibitors

Cells were seeded into 96-well plates (UWB1.289 2000 cells/well; UWB1.289+BRCA1 1000 cells/well; PEO1 1500 cells/well; PEO4 4000 cells/well; OVCAR-3 3000 cells/well; A2780 and A2780veliR 5000 cells/well) and treated with niraparib, olaparib, rucaparib, talazoparib (cat. #HY-10619, cat. #HY-10619, cat. #HY-10617, cat. #HY-16106, respectively; MedChemExpress, Monmouth Junction, NJ, USA) or veliparib (ABT-888; cat. #ALX-270-444-M005, Sapphire Biosciences, Waterloo, NSW, Australia) for 5 days before being assessed for cell viability using the CellTiter 96 Aqueous One Solution Cell Proliferation Assay (cat. #G3581, Promega, Madison, USA). This assay measures cellular metabolic activity and is a surrogate for cell viability. All results using this assay are described in the context of cell viability. Each experiment was performed in triplicate and repeated four times, with data reported as the mean ± SEM.

Relative lethal concentration 50 (LC50), the concentration required to bring the dose curve halfway between the top and bottom plateau of the curve, was calculated using GraphPad Prism 9. To address the issue that some of the drugs tested did not achieve total loss of cell viability at high levels, data were normalised to vehicle alone (100%) and the highest drug concentration where a plateau was observed (0%). A non-linear regression curve was fitted to the data and LC50 concentrations extrapolated [57].

### 4.4. Clonogenic Cell Survival Analyses

Clonogenic cell survival assays measure the ability of a single cell to grow into a colony post an intervention, in this case treatment with a PARP inhibitor or down-regulation of a gene. All results using this assay are described in the context of cell survival. Cells were seeded into 6-well plates at a density of 1000 cells/well for UWB1.289+BRCA1 and OVCAR-3, 2000 cells/well for UWB1.289, 750 cells/well for PEO1, 1500 cells/well for PEO4, 200 cells/well for A2780 and 180 cells/well for A2780veliR. Cells were then treated with niraparib, olaparib, rucaparib, talazoparib or veliparib for 8–21 days. Cells were fixed with 100% methanol for 20 min, rinsed briefly with water and stained with 0.5% *w/v* crystal violet in 25% *v/v* methanol for 5 min [58]. Colonies were counted using the GelCount imager (Oxford Optronix, Abingdon, England) and plating efficiency (PE) and surviving fraction (SF) determined [59]. A Jupyter notebook script was written in Anaconda 3 and LC50 concentrations determined based on a published method for analysis of dose-survival curves [60].

### 4.5. Statistical Analysis

IBM SPSS software version 27.0 (SPSS Australasia Pty Ltd., Chatswood, NSW, Australia) was used for statistical analyses. All results are expressed as the mean ± SEM from at least three independent experiments unless otherwise stated. One-sample t-tests were used to assess the efficacy of gene down-regulation. Independent samples T tests were used to compare plating efficiency in cell survival assays. Two-way ANOVA were used to compare paired cell lines over dose courses for different PARPis. One-way ANOVA with Tukey’s *post hoc* test was used to test for multiple comparisons between cell lines at discrete drug dosages. For all analyses, *p* < 0.05 was considered statistically significant.

## Figures and Tables

**Table 1:** LC50 comparisons (cell proliferation; MTS) for PARP is in *BRCA1/2*–WT paired HGSOC cell lines and a parental–veliparib resistant endometrioid ovarian cancer cell line.

	LC50 (µM)		^ Fold Change
**PARPi**			
**PEO1 vs. PEO4**			
	**PE01**	**PE04**	
niraparib	1.9300	5.0640	2.62
rucaparib	2.6370	3.2140	1.22
olaparib	2.3560	2.5710	1.09
talazoparib	0.0729	0.0557	0.76
veliparib	47.5900	28.1300	0.59
**UWB1.289 vs. UWB1.289 + BRCA1**			
	**UWB1.289**	**UWB1.289 + BRCA1**	
niraparib	0.6936	2.4620	3.55
rucaparib	2.2560	7.9120	3.51
veliparib	4.8490	15.7200	3.24
olaparib	0.1679	0.4920	2.93
talazoparib	0.0025	0.0062	2.52
**A2780 vs. A2780VeliR**			
	**A2780**	**A2780VeliR**	
talazoparib	0.0024	0.0347	14.41
rucaparib	1.1440	6.3480	5.55
**veliparib**	**16.62**	**57.49**	**3.46**
niraparib	0.2934	0.8718	2.97
olaparib	0.8735	2.2230	2.54

^ fold change is displayed in descending order.

**Table 2:** LC50 comparisons (cell survival; clonogenic assay) for PARP is in *BRCA1/2*–WT paired HGSOC cell lines and a parental–veliparib resistant endometrioid ovarian cancer cell line.

	LC50 (µM)		^ Fold Change
**PARPi**			
**PEO1 vs. PEO4**			
	**PEO1**	**PEO4**	
rucaparib	0.0417	0.5332	12.78
niraparib	0.2168	0.9263	4.27
olaparib	0.1405	0.4935	3.51
talazoparib	0.00008	0.00017	2.23
veliparib	4.445	8.2154	1.85
**UWB1.289 vs. UWB1.289 + BRCA1**			
	**UWB1.289**	**UWB1.289 + BRCA1**	
veliparib	0.1745	8.2640	47.36
rucaparib	0.0066	0.2592	39.30
olaparib	0.0091	0.2446	26.77
niraparib	0.0178	0.1889	10.63
talazoparib	0.00005	0.00026	4.86
**A2780 vs. A2780VeliR**			
	**A2780**	**A2780VeliR**	
olaparib	0.0052	0.1206	23.03
rucaparib	0.0071	0.0915	12.97
veliparib	0.5395	4.7707	8.84
talazoparib	0.00003	0.0001	2.99
niraparib	0.0194	0.0484	2.49

^ fold change is displayed in descending order.

**Table 3:** LC50 comparisons (cell survival; clonogenic assays) for PARPis in cell lines with down-regulated *BRCA1.*

	LC50 (µM)		
	AS	*BRCA1* si#13 (fold change)	*BRCA1* si#14 (fold change ^)
**PARPi**			
**A2780 *BRCA1* KD (cell viability)**			
olaparib	1.2330	0.1994 (6.18)	0.3539 (3.48)
rucaparib	0.8272	0.1886 (4.39)	0.3320 (2.49)
talazoparib	0.0009	0.0003 (3.32)	0.0004 (2.32)
veliparib	16.9900	5.3730 (3.16)	8.0910 (2.10)
niraparib	0.4010	0.1910 (2.10)	0.1927 (2.08)
**OVCAR-3 *BRCA1* KD (cell viability)**			
talazoparib	0.0058	0.0006 (10.24)	0.0005 (11.81)
rucaparib	2.4500	0.1345 (18.22)	0.2293 (10.68)
niraparib	3.6650	0.4730 (7.75)	0.3468 (10.57)
olaparib	2.9110	0.4229 (6.88)	0.4687 (6.21)
veliparib	20.5600	3.2850 (6.26)	3.6540 (5.63)
**A2780 *BRCA1* KD (cell survival)**			
olaparib	0.0275	0.0075 (3.67)	0.0110 (2.50)
niraparib	0.0471	0.0204 (2.30)	0.0311 (1.51)
veliparib	1.1726	0.5215 (2.25)	0.8487 (1.38)
talazoparib	0.0006	0.0003 (2.10)	0.0004 (1.26)
rucaparib	0.0283	0.0119 (2.38)	0.0229 (1.24)
**OVCAR-3 BRCA1 KD (cell survival)**			
rucaparib	0.0173	0.0085 (2.04)	0.0074 (2.33)
veliparib	1.5195	1.3455 (1.13)	0.7451 (2.04)
olaparib	0.0089	0.0062 (1.43)	0.0056 (1.59)
niraparib	0.0319	0.0263 (1.22)	0.0206 (1.55)
talazoparib	0.0003	0.0003 (0.94)	0.0003 (0.93)

^ fold change is displayed in descending order for BRCA1 si#14; KD, knock-down; AS, AllStars non-silencing control.

## Data Availability

All data presented in this study are linked through the University of Technology Sydney’s research data management platform Stash and is available upon request to the corresponding author.

## Supplementary Materials

The following are available online at https://www.mdpi.com/article/10.3390/ijms22168506/s1.
