# Supplementary material for: PARP Inhibitors Display Differential Efficacy in Models of BRCA Mutant High-Grade Serous Ovarian Cancer
Source: Int J Mol Sci. 2021 Aug 7;22(16):8506. doi: 10.3390/ijms22168506 (PMC8395221; doi:10.3390/ijms22168506)
Supplement: Supplementary file 1 [file ijms-22-08506-s001.zip › ijms-1318562-supplementary.pdf]

SUPPLEMENTARY MATERIAL

PARP inhibitors display differential efficacy in models of *BRCA* mutant high-grade serous ovarian cancer

Kristie-Ann Dickson[1,*], Tao Xie[1,*], Christian Evenhuis[2], Yue Ma[1] and Deborah J Marsh[1,3,^].

[1]Translational Oncology Group, School of Life Sciences, Faculty of Science, University of Technology Sydney, Ultimo, NSW 2007, Australia; [2]iThree Institute, School of Life Sciences, Faculty of Science, University of Technology Sydney, Ultimo, NSW 2007, Australia; [3]Northern Clinical School, Faculty of Medicine and Health, University of Sydney, Camperdown, NSW 2006, Australia.

**contributed equally to this work*

SUPPLEMENTARY METHODS

Methods S1: Description of the script used for analyses of cell survival data collected from clonogenic assays.

The model

The dose-response relationship was modelled using generalised logistic function of the log-concentration scale:

$$gl(c; PE, \phi, LC50, slope, \nu) = PE\left(\frac{1-\phi}{[1+\exp(slope(\ln(c)-\ln(LC50))]} + \phi\right)$$

The four parameters in the equations are:

PE	**Plating efficiency** – the percentage of cells that adhere to the plate after transfer.
ϕ	**Non-responsive fraction** – the percentage of cells that are not affected by the drug. This includes any debris or imperfections in the gel which get falsely identified as clusters of cells by the image detection step.

$LC50$	**Lethal Concentration 50** – the concentration of drug at which half of colonies that respond to the treatment are inhibited.
$slope$	**Slope of the curve at the LC50** – the concentration of drug at which half of colonies that respond to the treatment

This functional form was chosen as it has a sigmoid shape and greater flexibility than the Linear-Quadratic (LQ) model. Unkel[1] demonstrated that the generalised logistic better described the response of clonogenic survival to ionising radiation than the simpler LQ model. The shape of generalised logistic function is similar to the Hill equation which is widely used to model dose-response relationships in pharmacology and toxicology.

Parameter Estimation

The values for the parameter were estimated in a Bayesian framework. Briefly, in a Bayesian analysis the variables (in our case $\Theta = [PE, \phi, LC50, slope, v]$) are viewed probabilistically. The probability of a variable having a given value given the observed data (**posterior** probability) is proportional to the probability of observation given the parameter values (**likelihood**) times the **prior** probability of the parameter values:

$$p(\Theta|obs) \propto \mathcal{L}(obs|\Theta) \times \pi(\Theta)$$

To setup a Bayesian analysis we needed to specify the likelihood function and the prior probabilities for each parameter. For a given cell line, for concentration of drug of c_i, the number of colonies observed on the plate n_i^{obs} is modelled as a Poisson count

$$\mathcal{L} = Possion\left(n_i^{obs}; \text{cells\_plated} \times gl(c_i; PE, \phi, LC50, slope)\right),$$

where gl is the generalised logistic equation described above.

The parameters were estimated in two ways. First with an un-pooled model in which each experimental run was estimated independently of one another, then in a hierarchical model. In

the hierarchical model a common term (or fixed effect) for cell line was included in the plating efficiency and for the LC50 and slope common terms for cell line and drug were included.

$$PE_{c,r} = PE_c + \delta PE_{c,d,r}$$

$$LC50_{c,d,r} = LC50_d + \delta LC50_c + \delta LC50_{c,d,r}$$

$$slope_{c,d,r} = slope_c + \delta slope_d + \delta slope_{c,d,r}$$

The residual term (or mixed effect), that is the part not described by the common or fixed term, were taken from a common distribution for all experimental runs. The variation in the estimates were reduced by enabling the information to be shared across experimental runs.

SUPPLEMENTARY TABLE

Supplementary Table S1: Concentrations and serial dilutions for all PARPis and specific cell lines used.

A. MTS assays

PARPi^	Concentration (µM)	Serial dilution	Cell Line
Olaparib	75 - 0.01	3 - fold	PEO1; PEO4; UWB1.289; UWB1.289+BRCA1; A2780; A2780veliR; A2780 BRCA1 siRNA KD^^; OVCAR3 BRCA1 siRNA KD
Rucaparib	80-0.001	4 - fold	PEO1; PEO4; UWB1.289; UWB1.289+BRCA1; A2780; A2780veliR; A2780 BRCA1 siRNA KD; OVCAR3 BRCA1 siRNA KD
Veliparib	200 – 0.03	3 - fold	PEO1; PEO4; UWB1.289; UWB1.289+BRCA1; A2780 BRCA1 siRNA KD; OVCAR3 BRCA1 siRNA KD
	200 – 0.3	3 - fold	A2780; A2780veliR
Niraparib	100-0.01	3 - fold	PEO1; PEO4; UWB1.289; UWB1.289+BRCA1;
	100 – 0.002	4 - fold	A2780; A2780veliR; A2780 BRCA1 siRNA KD; OVCAR3 BRCA1 siRNA KD
Talazoparib	85 – 0.0002	5 - fold	PEO1; PEO4; UWB1.289; UWB1.289+BRCA1; OVCAR3 BRCA1 siRNA KD
	2 – 0.000008	6 - fold	A2780; A2780veliR
	2 – 0.0000001	6 - fold	A2780 BRCA1 siRNA KD

B. Clonogenic assays

PARPi	Concentration (µM)	Serial dilution	Cell Line
Olaparib	4 – 0.008	2 - fold	PEO1; PEO4;
	4 – 0.0002	3 - fold	UWB1.289; UWB1.289+BRCA1
	0.5 – 0.00003	3 - fold	A2780; A2780veliR; A2780 BRCA1 siRNA KD; OVCAR3 BRCA1 siRNA KD
Rucaparib	4 – 0.008	2 - fold	PEO1; PEO4
	4 – 0.0002	3-fold	UWB1.289; UWB1.289+BRCA1; A2780; A2780veliR; A2780 BRCA1 siRNA KD; OVCAR3 BRCA1 siRNA KD
Veliparib	60 – 0.003	3 - fold	PEO1; PEO4; UWB1.289; UWB1.289+BRCA1

	20 – 0.001	3 - fold	A2780; A2780veliR; A2780 BRCA1 siRNA KD; OVCAR3 BRCA1 siRNA KD
Niraparib	8 – 0.016	2 - fold	PEO1; PEO4;
	1 – 0.00005	3 - fold	UWB1.289; UWB1.289+BRCA1; A2780; A2780veliR; A2780 BRCA1 siRNA KD; OVCAR3 BRCA1 siRNA KD;
Talazoparib	0.001 – 0.00002	2 - fold	PEO1; PEO4; UWB1.289; UWB1.289+BRCA1; A2780; A2780veliR; A2780 BRCA1 siRNA KD; OVCAR3 BRCA1 siRNA KD

^PARPi, PARP inhibitor; ^^KD, knock-down

SUPPLEMENTARY FIGURES AND LEGENDS

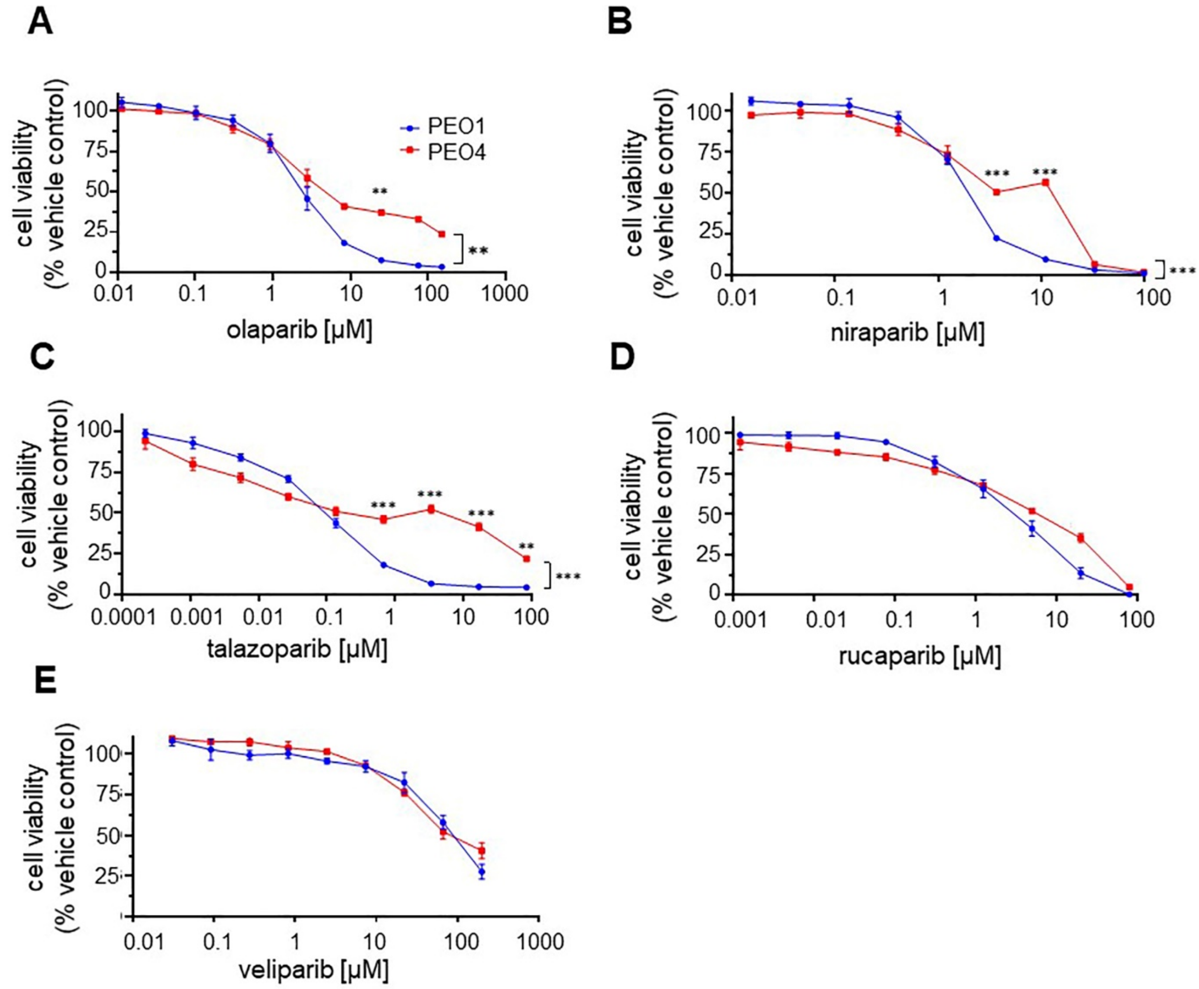

Figure S1: Dose curves in the PEO1 and PEO4 cell line pair measuring cell proliferation (MTS data) for ***A)*** olaparib ***B)*** niraparib ***C)*** talazoparib ***D)*** rucaparib ***E)*** veliparib. Two-way ANOVA was used to determine differences between cell lines over the dose curve. One-way ANOVA with Tukey *post hoc* test was used for multiple comparisons to determine significance at individual dose points. Each data point represents the experimental mean ± SEM (N = 3). ***P*<0.01, *** *P*<0.001.

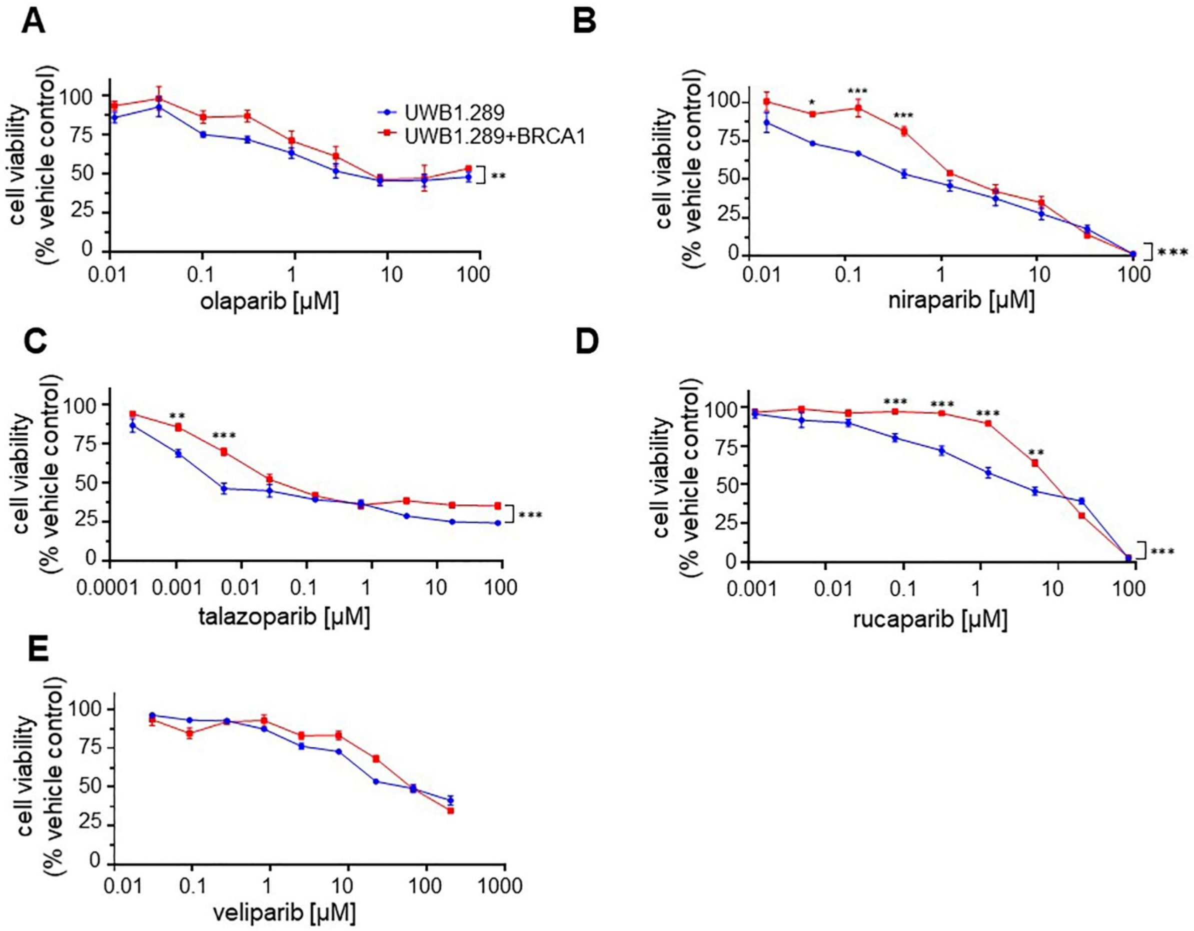

Figure S2: Dose curves in the UWB1.289 and UWB1.289+BRCA1 cell line pair measuring cell proliferation (MTS data) for ***A)*** olaparib ***B)*** niraparib ***C)*** talazoparib ***D)*** rucaparib ***E)*** veliparib. Two-way ANOVA was used to determine differences between cell lines over the dose curve. One-way ANOVA with Tukey *post hoc* test was used for multiple comparisons to determine significance at individual dose points. Each data point represents the experimental mean ± SEM (N = 3). * $P<0.05$, ** $P<0.01$, *** $P<0.001$.

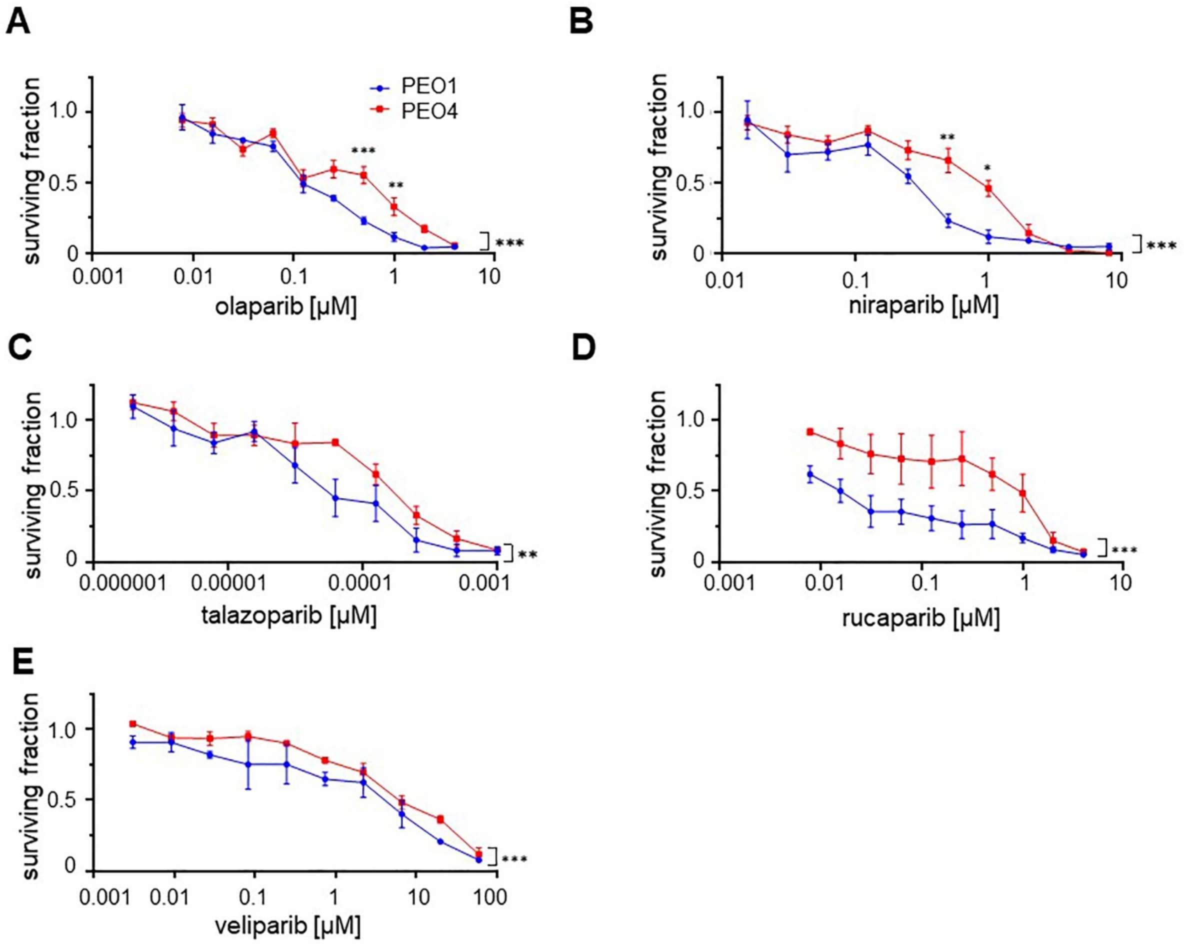

Figure S3: Dose curves in the PEO1 and PEO4 cell line pair measuring cell survival (clonogenic assay data) for ***A)*** olaparib ***B)*** niraparib ***C)*** talazoparib ***D)*** rucaparib ***E)*** veliparib. Two-way ANOVA was used to determine differences between cell lines over the dose curve. One-way ANOVA with Tukey *post hoc* test was used for multiple comparisons to determine significance at individual dose points. Each data point represents the experimental mean ± SEM (N = 3). * P<0.05, ** P<0.01, *** P<0.001.

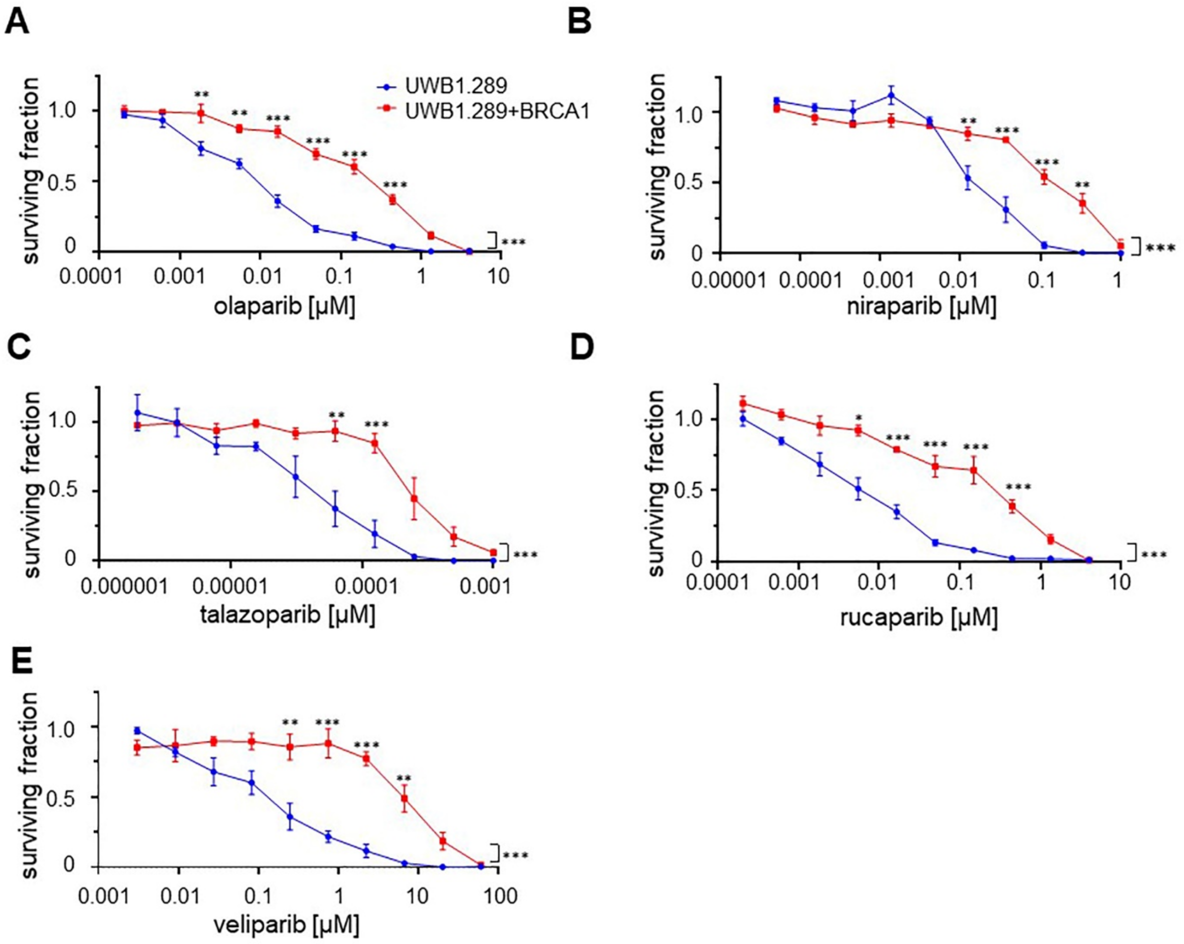

Figure S4: Dose curves in the UWB1.289 and UWB1.289+BRCA1 cell line pair measuring cell survival (clonogenic assay data) for ***A)*** olaparib ***B)*** niraparib ***C)*** talazoparib ***D)*** rucaparib (N = 4) ***E)*** veliparib. Two-way ANOVA was used to determine differences between cell lines over the dose curve. One-way ANOVA with Tukey *post hoc* test was used for multiple comparisons to determine significance at individual dose points. Each data point represents the experimental mean ± SEM (N = 3) unless otherwise stated. * $P<0.05$, ** $P<0.01$, *** $P<0.001$.

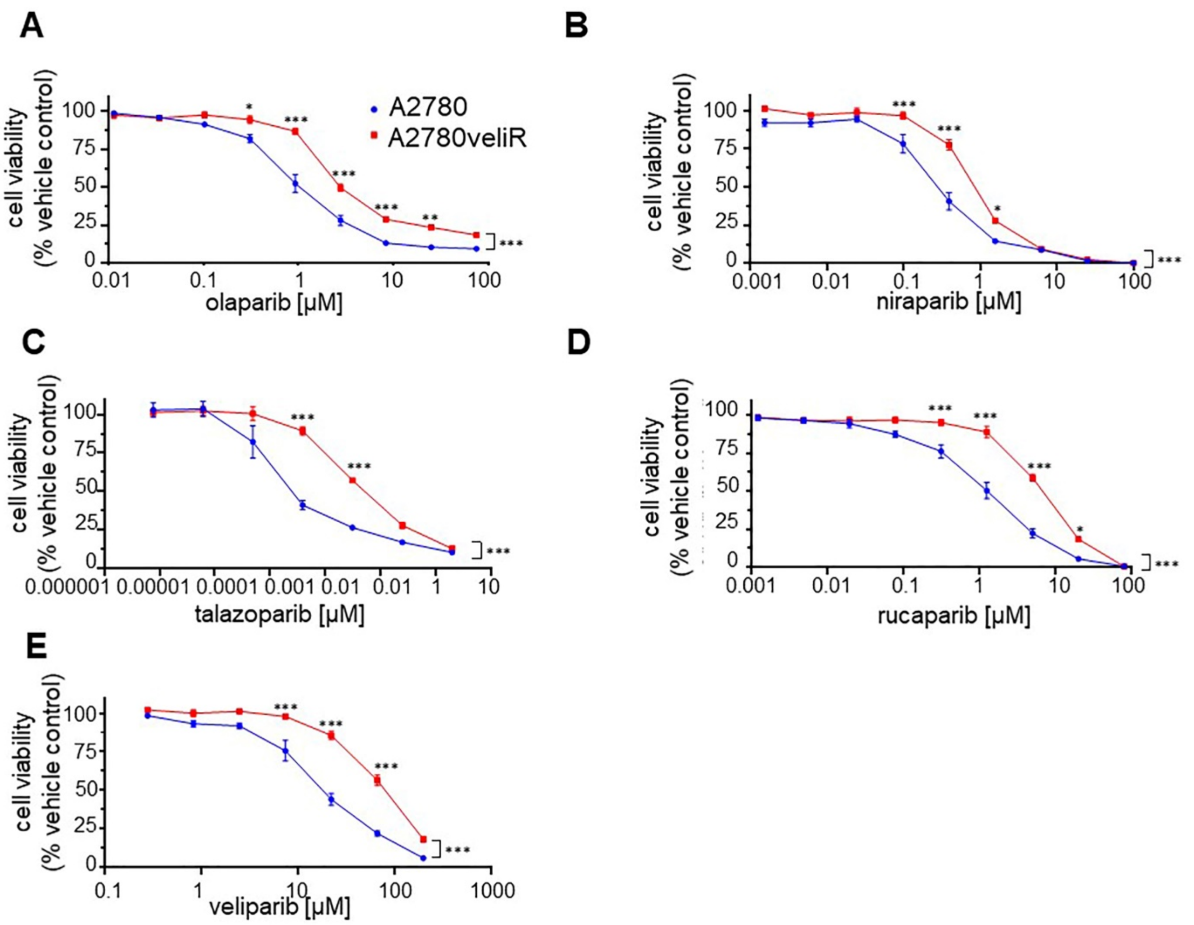

Figure S5: Dose curves in the A2780 and A2780veiR cell line pair measuring cell proliferation (MTS data) for ***A)*** olaparib ***B)*** niraparib ***C)*** talazoparib ***D)*** rucaparib ***E)*** veliparib. Two-way ANOVA was used to determine differences between cell lines over the dose curve. One-way ANOVA with Tukey *post hoc* test was used for multiple comparisons to determine significance at individual dose points. Each data point represents the experimental mean ± SEM (N = 3). * P<0.05, ** P<0.01, *** P<0.001.

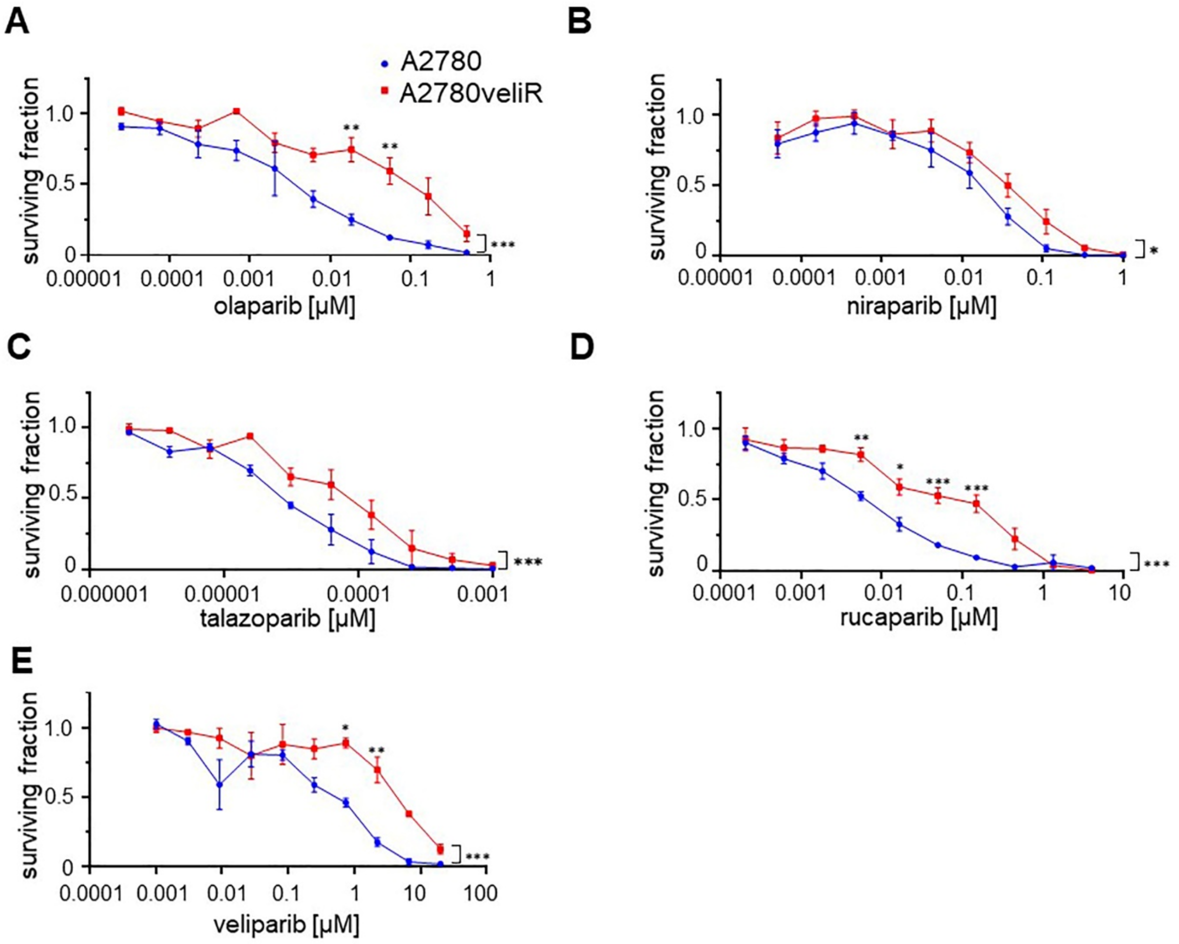

Figure S6: Dose curves in the A2780 and A2780veliR cell line pair measuring cell survival (clonogenic assay data) for ***A)*** olaparib ***B)*** niraparib ***C)*** talazoparib ***D)*** rucaparib ***E)*** veliparib. Two-way ANOVA was used to determine differences between cell lines over the dose curve. One-way ANOVA with Tukey *post hoc* test was used for multiple comparisons to determine significance at individual dose points. Each data point represents the experimental mean ± SEM (N = 3). * P<0.05, ** P<0.01, *** P<0.001.

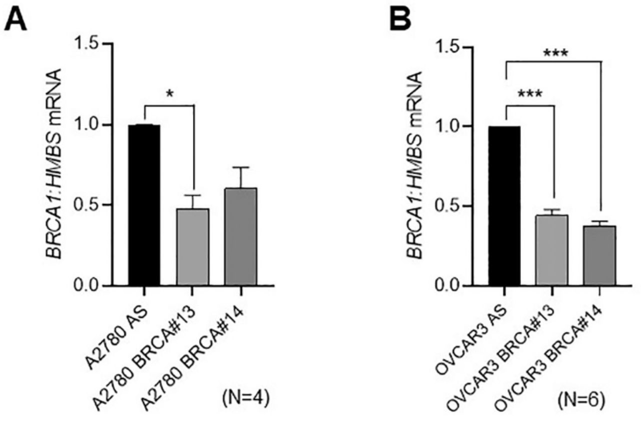

Figure S7: Efficacy of down-regulation of *BRCA1* by two independent siRNA (#13 and #14) in ***A)*** A2780 (N = 4) and ***B)*** OVCAR-3 cells (N = 6). Data was normalised to the reference gene *HMBS*, presented relative to the AS (Allstars) control and analysed using a one-sample t-test. * $P<0.05$, *** $P<0.001$.

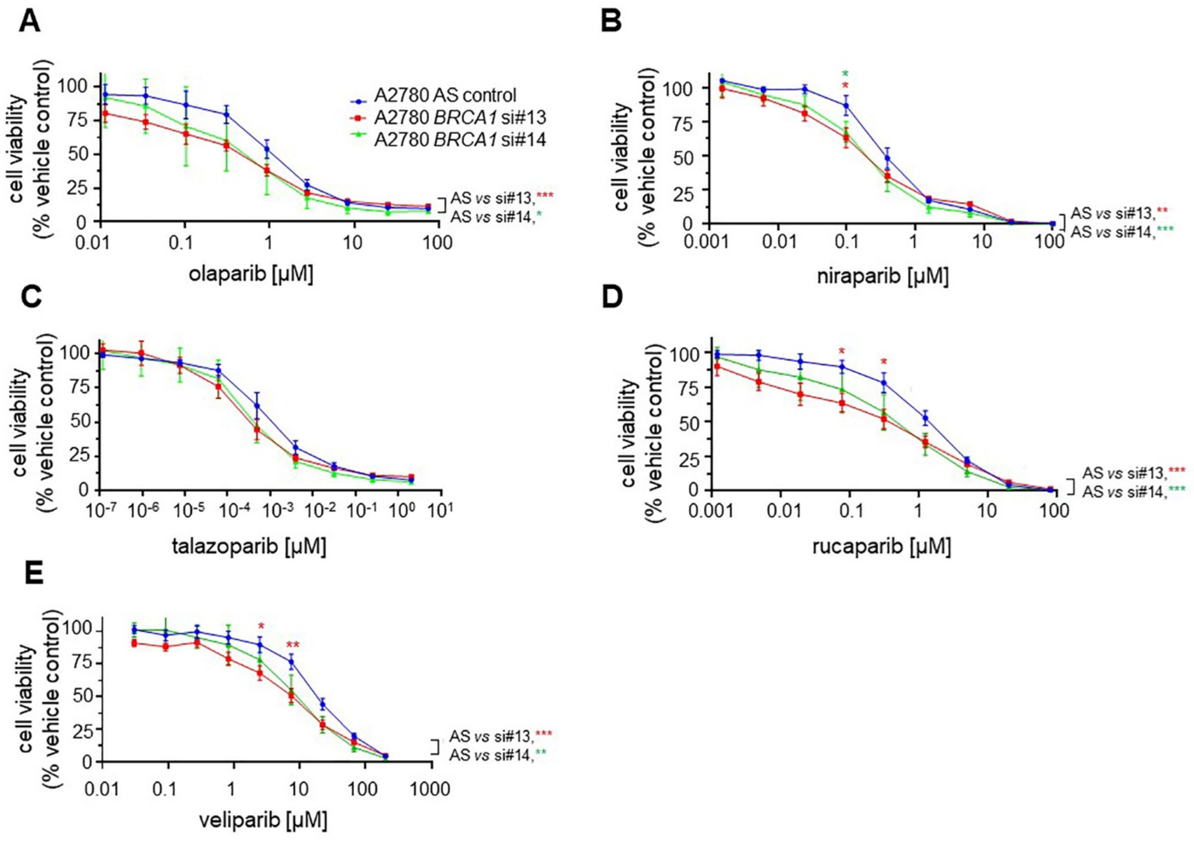

Figure S8: A2780 cell line dose curves measuring cell proliferation (MTS data) to determine whether down-regulation of two independent *BRCA1* siRNA (si#13 and si#14) sensitise cells to a PARPi compared to the AllStars (AS) non-silencing control: ***A)*** olaparib ***B)*** niraparib ***C)*** talazoparib ***D)*** rucaparib ***E)*** veliparib. Two-way ANOVA was used to determine differences between cell lines over the dose curve. One-way ANOVA with Tukey *post hoc* test was used for multiple comparisons to determine significance at individual dose points. Each data point represents the experimental mean ± SEM (N = 4) * $P<0.05$, ** $P<0.01$, *** $P<0.001$.

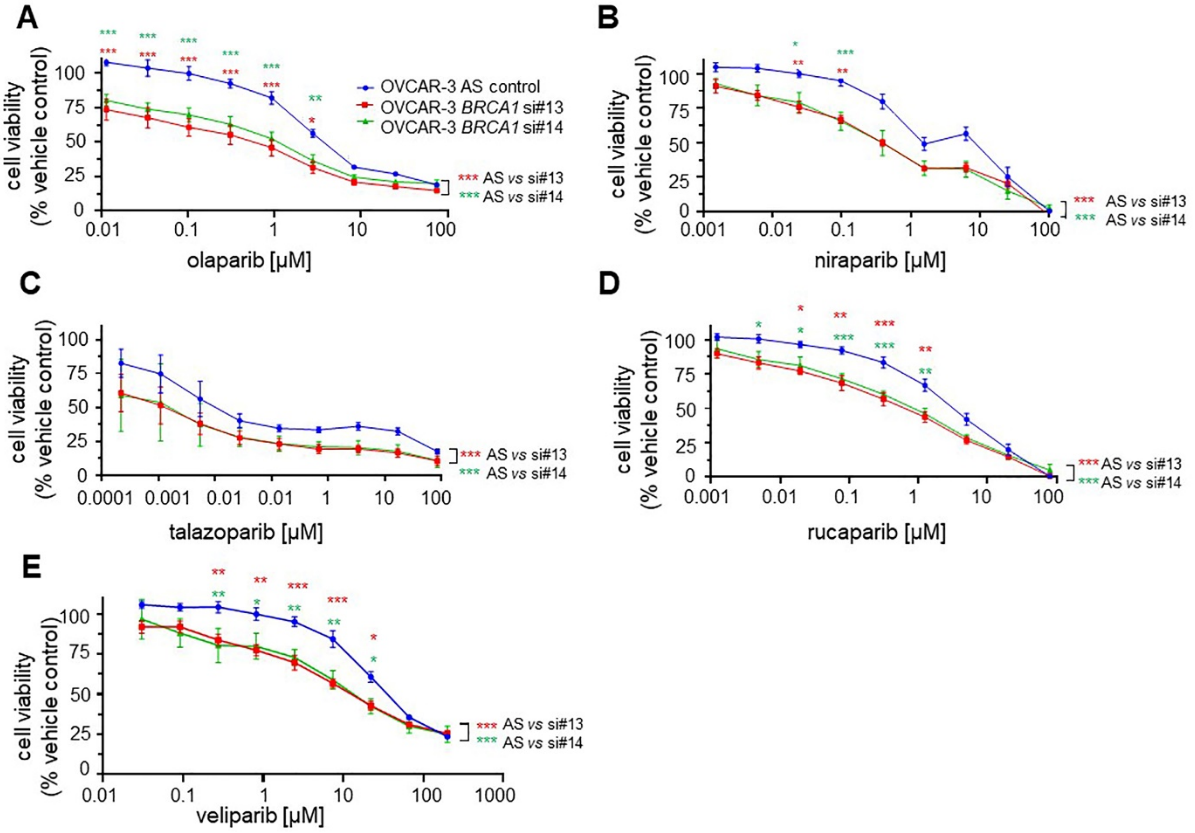

Figure S9: OVCAR-3 cell line dose curves measuring cell proliferation (MTS data) to determine whether down-regulation of two independent *BRCA1* siRNA (si#13 and si#14) sensitise cells to a PARPi compared to the AllStars (AS) non-silencing control: ***A)*** olaparib ***B)*** niraparib ***C)*** talazoparib ***D)*** rucaparib ***E)*** veliparib. Two-way ANOVA was used to determine differences between cell lines over the dose curve. One-way ANOVA with Tukey *post hoc* test was used for multiple comparisons to determine significance at individual dose points. Each data point represents the experimental mean ± SEM (N = 4) * $P<0.05$, ** $P<0.01$, *** $P<0.001$.

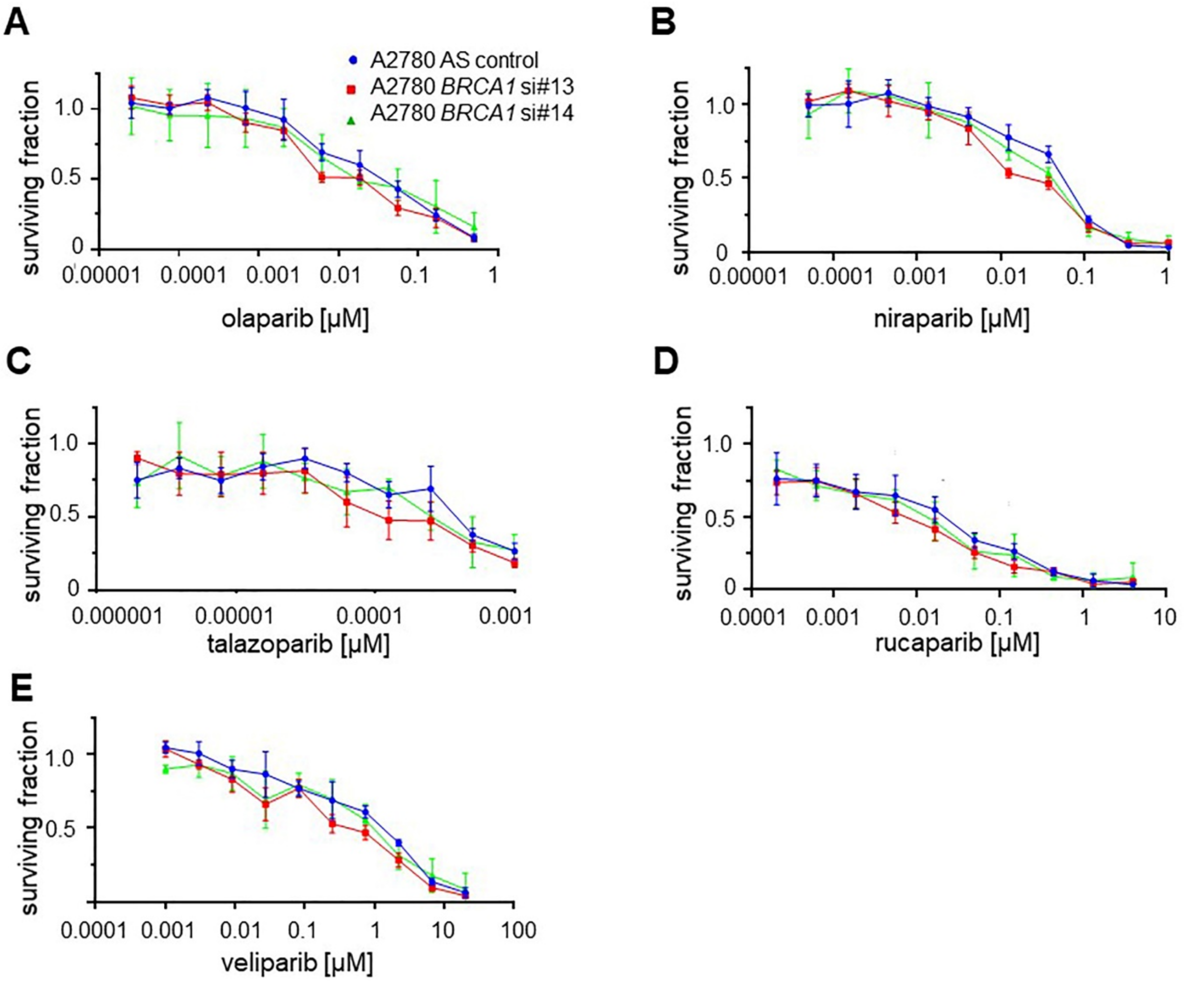

Figure S10: A2780 cell line dose curves measuring cell survival (clonogenic assay) to determine whether down-regulation of two independent *BRCA1* siRNA (si#13 and si#14) sensitise cells to a PARPi compared to the AllStars (AS) non-silencing control: ***A)*** olaparib (N=4) ***B)*** niraparib (N=4) ***C)*** talazoparib (N=3) ***D)*** rucaparib (N=4) ***E)*** veliparib (N=4). Two-way ANOVA was used to determine differences between cell lines over the dose curve. Each data point represents the experimental mean ± SEM. No significant difference was observed between cell line treatments.

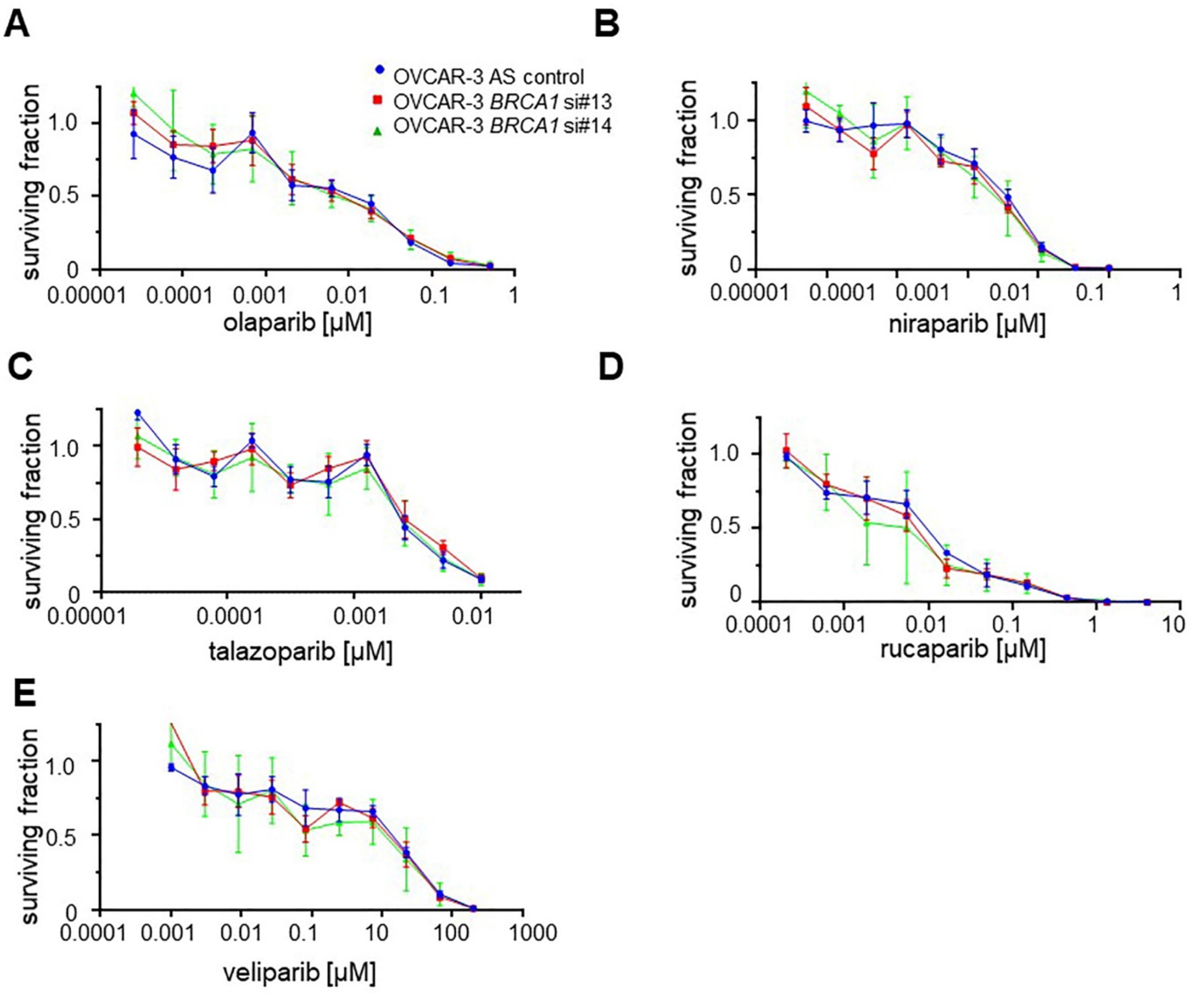

Figure S11: OVCAR-3 cell line dose curves measuring cell survival (clonogenic assay) to determine whether down-regulation of two independent *BRCA1* siRNA (si#13 and si#14) sensitise cells to a PARPi compared to the AllStars (AS) non-silencing control: ***A)*** olaparib (N=4) ***B)*** niraparib (N=3) ***C)*** talazoparib (N=4) ***D)*** rucaparib (N=3) ***E)*** veliparib (N=3). Two-way ANOVA was used to determine differences between cell lines over the dose curve. Each data point represents the experimental mean ± SEM. No significant difference was observed between cell line treatments.
